# Vaccinia virus protein C16 acts intracellularly to modulate the host response and promote virulence

**DOI:** 10.1099/vir.0.2008/004895-0

**Published:** 2008-10

**Authors:** Aodhnait S. Fahy, Richard H. Clark, Emily F. Glyde, Geoffrey L. Smith

**Affiliations:** Department of Virology, Faculty of Medicine, Imperial College London, St Mary's Campus, Norfolk Place, London W2 1PG, UK

## Abstract

The vaccinia virus (VACV) strain Western Reserve C16 protein has been characterized and its effects on virus replication and virulence have been determined. The *C16L* gene is present in the inverted terminal repeat and so is one of the few VACV genes that are diploid. The C16 protein is highly conserved between different VACV strains, and also in the orthopoxviruses variola virus, ectromelia virus, horsepox virus and cowpox virus. C16 is a 37.5 kDa protein, which is expressed early during infection and localizes to the cell nucleus and cytoplasm of infected and transfected cells. The loss of the *C16L* gene had no effect on virus growth kinetics but did reduce plaque size slightly. Furthermore, the virulence of a virus lacking *C16L* (vΔC16) was reduced in a murine intranasal model compared with control viruses and there were reduced virus titres from 4 days post-infection. In the absence of C16, the recruitment of inflammatory cells in the lung and bronchoalveolar lavage was increased early after infection (day 3) and more CD4^+^ and CD8^+^ T cells expressed the CD69 activation marker. Conversely, late after infection with vΔC16 (day 10) there were fewer T cells remaining, indicating more rapid clearance of infection. Collectively, these data indicate that C16 diminishes the immune response and is an intracellular immunomodulator.

## INTRODUCTION

*Vaccinia virus* (VACV) is the prototypical member of the genus *Orthopoxvirus* (OPV) of the *Poxviridae* and is famous as the live vaccine used to eradicate smallpox, an extinct human disease caused by variola virus (VARV) (Fenner *et al.*, 1988). Following the eradication of smallpox, VACV continues to be studied intensively because of its development as a vaccine vector (Panicali *et al.*, 1983; Smith *et al.*, 1983), and because of its fascinating interactions with the host cell and immune system (Seet *et al.*, 2003; Haga & Bowie, 2005). In this study, we have characterized the *C16L* gene of VACV strain Western Reserve (WR) and the encoded protein.

The VACV WR *C16L* gene is located in the inverted terminal repeat (ITR) at both ends of the VACV genome. Many genes within and adjacent to the ITRs encode proteins that modulate the host immune response or interactions with the host cell. For example, the gene upstream of *C16L* encodes the VACV growth factor (Twardzik *et al.*, 1985; Buller *et al.*, 1988), which promotes cell growth, and the gene two genes downstream encodes the interleukin (IL)-18-binding protein (Smith *et al.*, 2000; Symons *et al.*, 2002; Reading & Smith, 2003). The *C16L* gene is predicted to encode a 331 aa protein with a mass of 37.5 kDa (www.poxvirus.org). There are highly conserved orthologues of C16 in several other OPVs, suggesting an important function, and bioinformatic analysis identified a conserved 6 aa sequence at the C terminus of C16 that is present in the same region of the IL-1 receptor antagonist (IL-1ra) protein (Kluczyk *et al.*, 2002). This 6 aa sequence is also conserved in the VACV strain Copenhagen orthologue of C16 (termed C10) (Goebel *et al.*, 1990), and in orthologues in other OPVs (www.poxvirus.org). This sequence is critical for the ability of IL-1ra to antagonize signalling from the IL-1 receptor (IL-1R) and prompted the proposal that the VACV protein might act as a secreted viral IL-1ra and inhibit signalling from the IL-1R by receptor blockade (Kluczyk *et al.*, 2004).

The goals of this study were to characterize the C16 protein, determine its location and investigate if it affects virus replication or virulence. A VACV deletion mutant lacking both copies of the *C16L* gene (vΔC16) and a revertant virus (vC16Rev) in which the *C16L* gene was reinserted into the deletion mutant were constructed. Data presented show that C16 is non-essential for virus replication, but promotes VACV virulence in a murine intranasal (i.n.) model and delays the infiltration and activation of cells in the infected lungs. As such, it represents an intracellular virulence factor that functions as an immunomodulator.

## METHODS

### Cell culture.

BS-C-1, TK^−^143 and HeLa cells were grown at 37 °C in a 5 % CO_2_ atmosphere in Dulbecco's modified Eagle's medium supplemented with 10 % fetal bovine serum (FBS; Gibco). The source of VACV strain WR was described previously (Alcami & Smith, 1992).

### Construction of plasmid vectors.

A cassette containing the *Escherichia coli guanylphosphoribosyl transferase* (*Ecogpt*) gene fused in-frame with the *enhanced green fluorescent protein* (*EGFP)* gene and downstream of synthetic early/late VACV promoter was assembled as follows. The *Ecogpt* gene was amplified by PCR with primers AF1 (5′-AGTCGAATTCATAGCGAAAAATACATCGTCACCTGGGAC-3′) andAF2 (5′-GCGCTAGCGGATCTGAGCGACCGGAGATTGGCGGG-3′) and pGpt07/14 as template (Boyle & Coupar, 1988). The *EGFP* gene was amplified by PCR using primers AF3 (5′-CGCCAATCTCCGGTCGCTCAGATCCGCTAGCGCTACCG-3′) and AF4 (5′-AGTCCCCGGGATAAAAATTTACTTGTACAGCTCGTCCATGCCGAG-3′) and pEGFPC2 (BD Biosciences) as template. These PCR products were joined together by splice overlap extension (Horton *et al.*, 1989) by using primers AF1 and AF4. The product was digested with *Sma*I and *Eco*RI and cloned into pSEL (Bartlett *et al.*, 2004) downstream of a VACV synthetic early/late promoter (Davison & Moss, 1990) forming pSEL-Ecogpt/EGFP (also called pAFA).

The flanking regions of the *C16L* gene were amplified from VACV WR genomic DNA by using primers AF5 (5′-GACGGTATGTATTGTAGATGCTCTCATGG-3′) and AF6 (5′-CGTCAAACAATCATT*CCCGGG*TATAATATCTAGAGGTAGAGG-3′) for the 395 bp upstream region and primers AF7 (5′-CCTCTAGATATTATA*CCCGGG*AATGATTGTTTGACGAATCACG-3′) and AF8 (5′-GGAGATCATACTACCACAACTTATTATTATGC-3′) for the 383 bp downstream flanking region. These PCR products were ligated together using a *Sma*I site (indicated in italics) and then cloned into pGEMT by restriction digestion with *Eco*RI and *Not*I to form pGEMT-ΔC16L plasmid (pAFC).

The cassette containing pSEL driving Ecogpt/EGFP was excised from pAFA with *Sma*I and ligated into *Sma*I-digested pAFC to place the Ecogpt/EGFP cassette between the *C16L* flanking regions. This resultant plasmid, pGEMT-ΔC16L-Ecogpt/EGFP (also called pAFB) was used to construct the *C16L* deletion virus. To make a revertant virus, in which the *C16L* gene was reinserted into its natural loci, the *C16L* gene and flanking regions were amplified from VACV strain WR genomic DNA by using primers AF5 and AF8. The product was digested with *Eco*RI and *Not*I, ligated into pGEMT that had been digested with the same enzymes and termed pAFD.

The fidelity of all PCR-generated DNA sequences was confirmed by sequencing.

### Construction of recombinant viruses.

A VACV deletion mutant wherein both copies of the *C16L* gene were replaced with the Ecogpt/EGFP cassette was constructed by transfecting pAFB into CV-1 cells that had been infected with a plaque purified VACV WR (vC16). Recombinant viruses were selected in the presence of mycophenolic acid, xanthine and hypoxanthine (Boyle & Coupar, 1988). These recombinant viruses were also screened for EGFP expression by fluorescent microscopy and, after further rounds of plaque purification on BS-C-1 cells, were analysed by PCR to ensure replacement of both copies of the *C16L* gene with the Ecogpt/EGFP cassette. The resultant plaque-purified virus was called vΔC16/Ecogpt/EGFP.

To remove the Ecogpt/EGFP cassette from the *C16L* locus, plasmid AFC was transfected into vΔC16/Ecogpt/EGFP-infected cells and Ecogpt-negative viruses were selected using 6-thioguanine on D-98OR cells (Kerr & Smith, 1991). The deletion mutant virus was called vΔC16. A revertant virus was constructed in a similar manner by transfection of plasmid AFD into vΔC16/Ecogpt/EGFP-infected cells to create vC16Rev. Viruses were analysed by PCR using primers AF5 and AF8 to characterize the *C16L* locus. Genomic DNA was also analysed by restriction enzyme digestion and electrophoresis on a 0.6 % agarose gel.

### Expression of C16 in *E. coli* and production of anti-C16 rabbit serum.

The C16 ORF was amplified by PCR using primers AF9 (5′-AGAT*CCATGG*GTGATATTTACGACGATAAAGGTCTACAG-3′) and AF10 (5′-AGAT*GAATTC*CCGCTGCCGCGCGGCACCAGTTTCGGCATATTAAAGTAAAATC-3′) and VACV WR DNA as template. The product was digested with *Nco*I and *Eco*RI (indicated in italics) and cloned into *Nco*I- and *Eco*RI-digested pET28a (Novagen) and the resultant plasmid, pET28a-C16L-CHis, was termed pAFE. Recombinant C16 protein was expressed from *E. coli* strain BL21 according to the vector manufacturer's instructions (Novagen) and was purified by nickel chelate and ion-exchange chromatography as described for the A41 protein (Ng *et al.*, 2001). The purified protein (100–200 μg with an estimated purity of 90 %) was injected into New Zealand White rabbits to produce anti-C16 serum (Harlan Sera Laboratories).

### Immunoblotting.

Cells were infected with the indicated viruses at 10 p.f.u. per cell and cell lysates and supernatants were prepared as described previously (Bartlett *et al.*, 2002). Antibodies used included rabbit polyclonal sera against VACV proteins C16 (1 : 2000) (see above), A41 (1 : 1000) (Ng *et al.*, 2001) and A56 (1 : 1000), or mouse monoclonal antibody AB1.1 against D8 (1 : 1000) (Parkinson & Smith, 1994). Secondary antibodies and detection systems were as described previously (Bartlett *et al.*, 2002).

### Cell fractionation.

BS-C-1 cells were infected at 0.1 p.f.u. per cell for 48 h, scraped from the dish and collected by centrifugation (800 ***g***, 5 min). The cell pellet was resuspended in hypotonic buffer (10 mM Tris/HCl, pH 9.0) and disrupted by Dounce homogenization. The nuclei were removed by centrifugation (300 ***g***, 5 min) and the cytoplasmic fraction (supernatant) was removed. Nuclei were washed four times in 10 mM Tris/HCl, pH 9 before incubation in RIPA buffer for 10 min. The mixture was centrifuged (1500 ***g***, 10 min) and the supernatant retained as the nuclear fraction.

### Immunofluorescence.

HeLa cells were grown on sterilized glass coverslips (borosilicate glass; BDH) in six-well plates and infected at 5 p.f.u. per cell. Where indicated, 1 ng leptomycin B (Sigma-Aldrich) ml^−1^ was added to the cells for 4 h (Wolff *et al.*, 1997). Cells were washed, fixed, permeablised and blocked as described previously (Chen *et al.*, 2006). Cells were stained with anti-C16 (1 : 100) or anti-p65 (1 : 50; Santa Cruz) at room temperature for 1 h, followed by secondary antibody staining and mounting as described previously (Chen *et al.*, 2006).

### Virus growth curves.

Monolayers of BS-C-1 cells were infected with either 10 or 0.01 p.f.u. per cell for measurement of one-step or multi-step growth kinetics, respectively, as described previously (Chen *et al.*, 2006).

### Murine i.n. and intradermal models of infection.

Female BALB/c mice (*n*=5, 6–8 weeks old) were infected i.n. with 5×10^3^ p.f.u. and monitored as described previously (Williamson *et al.*, 1990; Alcami & Smith, 1992). Female C57BL/6 mice (*n*=5, 6–8 weeks old) were inoculated intradermally (i.d.) in the ear pinnae with 1×10^4^ p.f.u. as described previously (Tscharke & Smith, 1999; Tscharke *et al.*, 2002).

### Analysis of cell populations of infected organs.

Mice were infected i.n. with 1×10^4^ p.f.u. and at the indicated time post-infection (p.i.) were sacrificed, and the bronchial alveolar lavage (BAL) and lung tissue were processed as described previously (Clark *et al.*, 2006; Jacobs *et al.*, 2006). Live cells in BAL or lung samples were counted, washed with FACS buffer (0.1 % BSA, 0.1 % NaN_3_ inz PBS), blocked and stained with appropriate combinations of fluorescein isothiocyanate-, phycoerythrin- or tricolour-labelled antibodies. These were grouped into anti-CD25, anti-CD69, anti-CD3, anti-CD8, anti-CD4 for T lymphocytes, anti-Ly6G on neutrophils, anti-DX5 on natural killer cells, anti-F480 on macrophages and the relevant isotype controls (BD biosciences) as described previously (Jacobs *et al.*, 2006). The presence of cell-surface markers was determined on a FACScan flow cytometer with CellQuest software (BD Biosciences). A lymphocyte gate was used to analyse data from at least 20 000 events.

### Murine vaccination and challenge analysis.

Female BALB/c mice (*n*=5, 6–8 weeks old) were vaccinated i.d. in the ear pinnae with 1×10^4^ p.f.u. of the indicated virus, and, 28 days later, were challenged i.n. with 5×10^6^ p.f.u. of VACV WR and monitored as described previously (Clark *et al.*, 2006).

### Statistical analysis.

Student's *t*-test (two tailed, unpaired) was used to examine the significance of raw data.

## RESULTS

### Construction of *C16L* deletion mutant and revertant viruses

To study the function of the C16 protein, a VACV strain WR mutant lacking both copies of the *C16L* gene, vΔC16, was constructed (Methods) from plaque-purified VACV WR (vC16). The isolation of this virus demonstrated that the *C16L* gene was non-essential for virus replication. A revertant virus in which the *C16L* gene was reinserted into both ITRs, vC16Rev, was also constructed. PCR using primers for the *C16L* gene locus confirmed the presence of the *C16L* gene in vC16 and vC16Rev, and its absence in vΔC16 (Supplementary Fig. S1 available in JGV Online). Analysis of genomic DNA by restriction enzyme digestion and agarose gel electrophoresis showed that the only discernible change between the viruses was caused by alteration to the *C16L* loci (data not shown).

### Analysis of C16 expression

**Fig. 1. f1:**
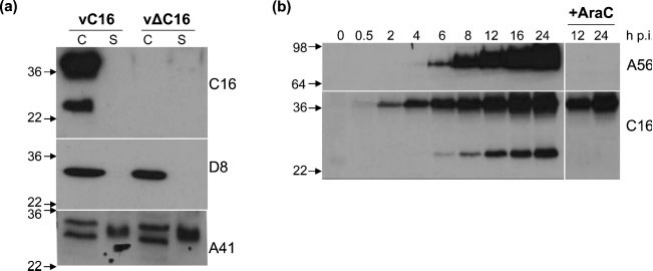
Characterization of C16 expression. BS-C-1 cells were infected with vC16 (a and b) or vΔC16 (a) in the presence (+) of 40 μg AraC ml^−1^ where indicated (b). Samples were analysed by SDS-PAGE and immunoblotting with antibodies against the indicated proteins. (a) Infected cell supernatants were removed at 16 h p.i., concentrated and equivalent amounts of cell lysate (C) and supernatant (S) were analysed by immunoblotting. (b) Cell lysates were prepared at the indicated times p.i. The positions of molecular mass marker are shown in kDa.

**Fig. 2. f2:**
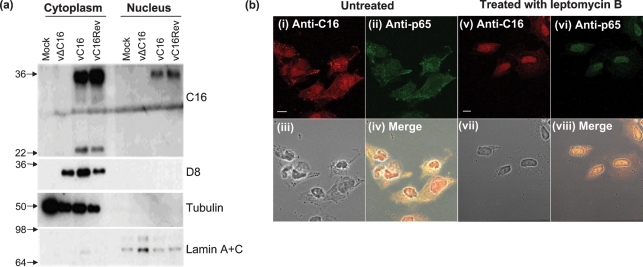
Localization of C16 in infected cells. (a) Immunoblotting. BS-C-1 cells were infected with the indicated viruses for 16 h and nuclear and cytoplasmic fractions were analysed by immunoblotting with antibodies against the indicated proteins. The positions of molecular mass marker are shown in kDa. (b) Immunofluorescence. Cells were infected with vC16 for 16 h and then incubated in the presence or absence of leptomycin B for 4 h. The localization of C16 [(i) and (v)] and NF-*κ*B subunit p65 [(ii) and (vi)] were analysed. Phase-contrast [(iii) and (vii)] and merged images [(iv) and (viii)] are also shown. Bar, 5 μm.

Immunoblot analysis using anti-C16 (Methods) identified 25 and 37 kDa proteins in extracts of cells infected with vC16 but not with vΔC16 (Fig. 1a[Fig f1]) or in mock-infected cells (Fig. 2a[Fig f2]). These proteins were found in cell lysates and not in the concentrated culture supernatant, whereas the secreted protein A41 (Ng *et al.*, 2001) was present in the culture medium and D8, a 35 kDa intracellular mature virus (IMV) membrane protein (Niles & Seto, 1988), was in the cell lysates.

To determine when C16 is expressed during infection, cells were infected in the presence or absence of cytosine arabinoside (AraC), an inhibitor of viral DNA replication and therefore late protein expression, and extracts of cells were analysed by immunoblotting (Fig. 1b[Fig f1]). The 37 kDa C16 protein was detected from 2 h p.i., and at all times thereafter. Notably, it was expressed in the presence of AraC, indicating expression early during infection. In contrast, AraC blocked the expression of A56, a protein expressed predominantly late during infection (Brown *et al.*, 1991). Notably, the 25 kDa C16 protein was present only late during infection and its formation was ablated in the presence of AraC.

The intracellular localization of the C16 protein was examined by biochemical fractionation of cells followed by immunoblotting (Fig. 2a[Fig f2]) and immunofluorescence (Fig. 2b[Fig f2]). Immunoblotting showed that cellular proteins tubulin and lamin A+C were present in the cytoplasmic or nuclear fraction, respectively, as expected. In contrast, the 37 kDa C16 protein was in both nuclear and cytoplasmic fractions after infection with vC16 and vC16Rev, but, even after longer exposure of the film, the 25 kDa C16 protein localized exclusively to the cytoplasm. For comparison, the VACV protein D8 was only in the cytoplasm.

Immunofluorescence also showed that C16 localized to the cytoplasm and nucleus [Fig. 2b[Fig f2](i)]. There was minimal background immunofluorescence in vΔC16-infected cells (data not shown). The partial nuclear localization was investigated further using leptomycin B, an inhibitor of active nuclear export. In the presence of leptomycin B, the great majority of C16 and p65 were present in the nucleus [panels (v) and (vi), suggesting active transport rather than diffusion through the nuclear pores (Fig. 2b[Fig f2])]. This localization pattern and active transport were similar in C16-transfected cells (data not shown).

### Computational analysis of the *C16L* gene

**Fig. 3. f3:**
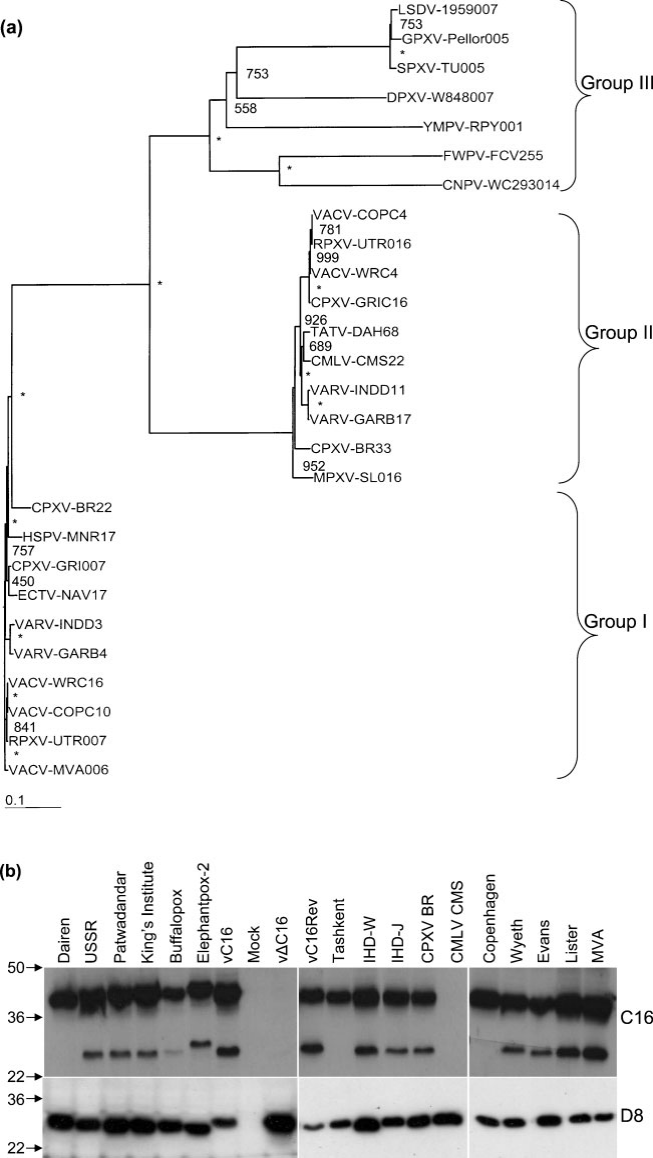
Conservation of C16. (a) A rooted phylogenetic tree of VACV WR C16 and related poxvirus proteins. Amino acid sequences from C16-like proteins (www.poxvirus.org) were aligned using the clustal w programme and a rooted tree was derived from this alignment using phylip (phylogeny inference package, version 3.67). The bootstrap values for 1000 replicate samplings are indicated, values of 1000 are indicated by *. Bar, Branch length of 0.1 substitutions per site. (b) BS-C-1 cells were infected with 14 VACV strains, two CPXV strains [Brighton Red, (BR) and elephantpox-2] and CMLV strain CMS. Cell lysates were prepared 16 h p.i., separated by SDS-PAGE and immunoblotted using antibody against C16 or D8. Molecular mass marker is shown in kDa.

The VACV WR *C16L* gene is present in both ITRs (GenBank accession nos YP_232892 and YP_233091) and is predicted to encode a protein without a transmembrane domain or signal peptide (www.poxvirus.org). Computational analysis found no cellular proteins with similarity to the VACV WR C16 protein, with the exception of the C-terminal peptide in the IL-1ra protein. However, C16 is highly conserved in several OPVs including variola virus (VARV), cowpox virus (CPXV), ectromelia virus (ECTV) and horsepox virus (HSPV). C16 is diploid in some strains of VACV, but not in other OPVs. In monkeypox virus (MPXV), camelpox virus (CMLV) and taterapox virus (TATV) the reading frame of the *C16L* orthologue is disrupted by mutation into shorter fragments. The phylogenetic relationships of these proteins are shown in a rooted tree (Fig. 3a[Fig f3]) produced from the aligned amino acid sequences. Group I proteins share 95–99 % amino acid identity and these proteins all have the C-terminal VTRFYF sequence, which is present in the IL-1ra protein (Kluczyk *et al.*, 2004).

Another group of more distantly related OPV proteins, typified by protein C4 from VACV WR, was identified and is shown as group II (Fig. 3a[Fig f3]). The C4 protein family is also conserved in several OPVs (VACV, VARV, CMLV, TATV, MPXV and CPXV) and these have 40–44 % amino acid identity to VACV WR C16. The degree of similarity between C16 and C4 is greatest in the C-terminal 100 aa, where these proteins share 55 % identity. Within the C4 group the proteins are highly conserved (95–99 % identity). Notably, C4 is encoded by CMLV, TATV and MPXV viruses that lack C16. The close similarity between the C4 and C16 proteins within OPVs suggest that they are likely to have been generated by a gene duplication event followed by diversification. The third group (III) of proteins (Fig. 3a[Fig f3]) comprises more distantly related proteins from other chordopoxviruses including goatpox virus (GPXV), lumpy skin disease virus (LSDV), sheeppox virus (all genus *Capripoxvirus*), fowlpox virus (genus *Avipoxvirus*), Yaba monkey tumor virus (genus *Yatapoxvirus*) and deerpox virus (DPXV) (unclassified). These proteins have 26–37 % amino acid identity (49–61 % similarity) to C16, and are closer to C16 than C4. Within the groups II and III proteins, the IL-1ra-like sequence, VTRFYF, is partially conserved with the consensus sequence VT(R/K)-Y-.

The expression of C16 by 14 VACV strains, two strains of CPXV and CMLV strain CMS was investigated by immunoblotting (Fig. 3b[Fig f3]). Protein(s) recognized by the anti-C16 serum were expressed by all 14 VACV strains and both strains of CPXV, but not by CMLV or vΔC16. The latter observation demonstrated that the anti-C16 antibody does not recognize the related C4 protein encoded by VACV strain WR. Infection with most viruses (but not VACV strains Dairen, Copenhagen and Tashkent) produced proteins of 37 and 25 kDa that were recognized by the anti-C16 antibody. These are both encoded by the *C16L* gene because they were absent in cells infected by vΔC16 and were expressed by vC16 and vC16Rev controls. The smaller polypeptide might be derived from the 37 kDa protein by proteolytic cleavage or (less likely) it might be generated by different translation initiation and its significance is unknown.

### C16 is non-essential in cell culture

**Fig. 4. f4:**
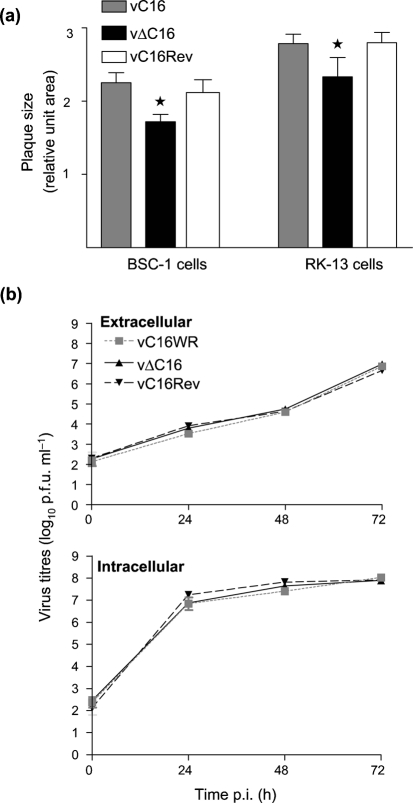
vΔC16 plaque formation and growth kinetics. (a) Plaque size. Monolayers of BS-C-1 or RK-13 cells were infected with the indicated viruses to give well separated plaques and at 72 h p.i. the size of 50 plaques was measured for each virus using Adobe Photoshop 7.0. Data are expressed as the average area (measured in pixels) (×10^5^)±sd. *=*P*<0.05 for vΔC16 compared with both vC16 and vC16Rev. (b) Growth curves. BS-C-1 cells were infected at 0.01 p.f.u. per cell and aliquots of culture medium (mainly extracellualr enveloped virus) or infected cells (mainly IMV) were collected at the indicated times p.i. The cells were frozen and thawed three times, sonicated and virus infectivity was titrated by plaque assay. Data are presented as the mean log_10_ p.f.u.±sd.

The isolation of vΔC16 demonstrated that C16 is not essential for VACV replication. To determine if deletion of *C16L* caused alterations in virus replication kinetics or spread, the growth of vΔC16 was compared with wild-type and revertant controls. Plaques formed by vΔC16 were slightly smaller (*P*<0.05) than controls in BS-C-1 and RK-13 cells (Fig. 4a[Fig f4]). To investigate if the reduced plaque size was due to reduced virus titres or reduced virus spread, the replication kinetics were analysed after low (0.01) or high (10) m.o.i. However, no difference was observed between vΔC16 and controls (Fig. 4b[Fig f4] and data not shown). Virus plaque formation is influenced by the production of actin tails beneath cell-associated enveloped virus particles on the cell surface (Smith *et al.*, 2002, 2003), and so the formation of actin tails by vΔC16 was analysed by confocal microscopy. However, no difference between vΔC16 and controls was observed (data not shown) and so the reason for the small difference in plaque size remains unknown.

### C16 affects virus virulence in the murine i.n. model

**Fig. 5. f5:**
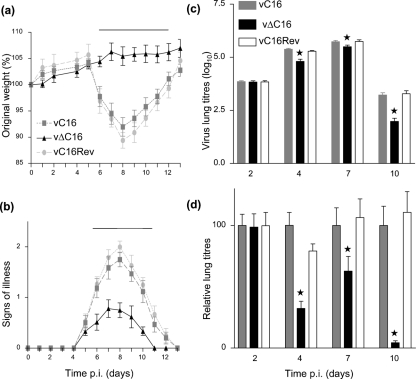
Virulence in murine i.n. model. BALB/c mice (*n*=5) were infected with the indicated viruses and their weights (a) and signs of illness (b) were measured daily (Methods). (a) Weights. Data are expressed as the percentage±sd of the mean weight of the same group of animals on day 0. (b) Signs of illness. The mean score±sem of each group of animals is shown. The horizontal bar indicates days on which the weight loss or signs of illness induced by vΔC16 was statistically different (*P*<0.05) from both vC16 and vC16Rev. (c and d) Virus titres in lungs. (c) Mice were infected i.n. as in (a) and, at the indicated times, groups (*n*=5) were sacrificed their lungs were removed and infectious virus per ml of lung homogenate was determined by plaque assay. (d) Data from (c) are presented as percentages of the virus titres in the vC16-infected lungs for that day±sd. *=*P*<0.05 for vΔC16 compared to both vC16 and vC16Rev.

The virulence of vΔC16 was compared with control viruses in two murine models. In an i.d. model, no significant difference in lesion size or cell recruitment was observed in animals infected with vΔC16 at 10^4^ p.f.u. compared to control viruses (Supplementary Fig. S2 available in JGV Online and unpublished data). However, in an i.n. model, vΔC16 induced significantly (*P*<0.05) less weight loss and milder signs of illness compared with controls (Fig. 5a and b[Fig f5]) between days 6 and 11. Moreover, although all viruses replicated initially to similar titres (day 2) there was significantly (*P*<0.05) less infectious virus in lungs infected by vΔC16 from day 4 p.i. onwards (Fig. 5c and d[Fig f5]). This accelerated virus clearance after infection with vΔC16 compared with controls suggested a more effective antiviral host response, and therefore the cellular inflammatory response in lungs was analysed by flow cytometry.

### C16 affects immune cell recruitment in the murine i.n. model

**Fig. 6. f6:**
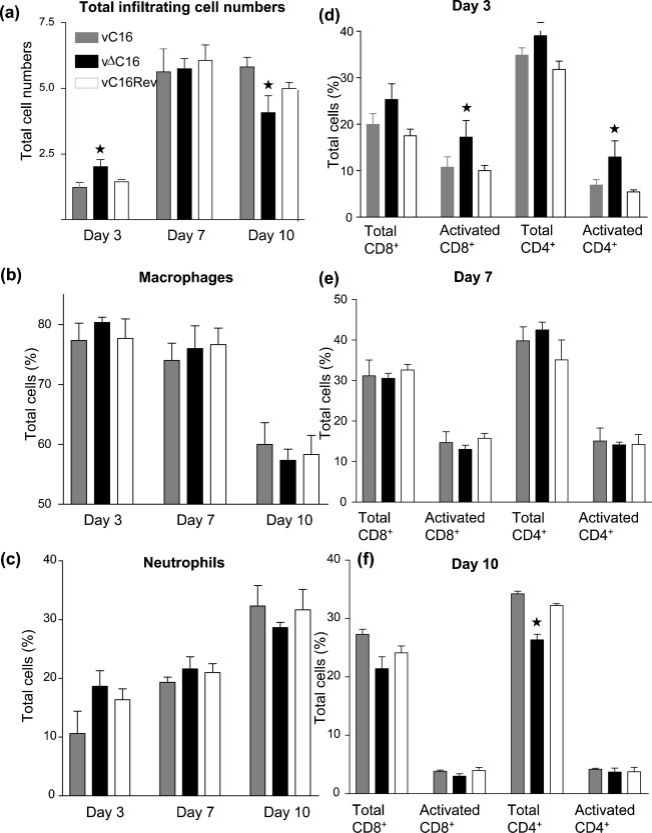
Characterization of infiltrating leukocytes. Cells were extracted from the BAL (a–c) and lungs (d–f) of infected mice (*n*=5), counted and analysed by flow cytometry. Asterisks indicate where data for vΔC16 are significantly different from the vC16 and vC16Rev (*P*<0.05, Student's *t*-test). (a) Total numbers of viable cells in the BAL. Data are means of cell counts±sd. (b and c) Macrophages (b) and neutrophils (c) were identified and quantified by FACS. (d–f). Lungs. The number and activation status (CD69^+^) of CD4^+^ and CD8^+^ T cells were analysed by FACS and are presented as a percentage of the total cells present.

Cells in infected lungs and BAL were extracted, quantified by trypan blue exclusion and identified by flow cytometry (Fig. 6a[Fig f6]). This revealed a statistically (*P*<0.05) higher number of cells in vΔC16-infected BALs at day 3 p.i., compared with controls. By day 7, the cell numbers had equalized between the viruses, whereas at day 10, there were fewer lymphocytes present in the BAL of mice infected with vΔC16. To test whether this early increase in lymphoid cells was due to an enhanced recruitment of a particular lymphoid subset, the percentage of macrophages (Fig. 6b[Fig f6]), neutrophils (Fig. 6c[Fig f6]), natural killer cells and CD4^+^ and CD8^+^ T lymphocytes (data not shown) present was analysed. This showed a slightly greater number of all cell types examined in the vΔC16-infected BAL at day 3, but there was no dramatic increase in any lymphoid subset.

Next, the recruitment and activation of T cells (CD3^+^) in the lungs was investigated by staining for CD3, CD4, CD8 and CD69 (Fig. 6d–f[Fig f6]). Natural killer cell (DX5^+^) recruitment in the lungs was also analysed and showed no significant variation between vΔC16 and control viruses (Supplementary Fig. S3 available in JGV Online). T-cell analysis showed that at day 3 more of the CD4^+^ and CD8^+^ T cells, recruited to the site of infection with vΔC16, were activated (CD69^+^) compared with controls. By day 7, there was no difference in the number of activated T lymphocytes after infection with the different viruses. However, by day 10 there was a reduction in CD4^+^ and CD8^+^ T cells present after infection with vΔC16. One interpretation of these data is that the host response to infection with vΔC16 is accelerated compared with controls, so that early p.i. there are more cells in vΔC16-infected tissues and these show a greater degree of activation, whereas at late times there are fewer cells because the infection is being cleared sooner. By inference, the C16 protein may function to diminish the innate response to infection.

### Vaccination and challenge with vΔC16

**Fig. 7. f7:**
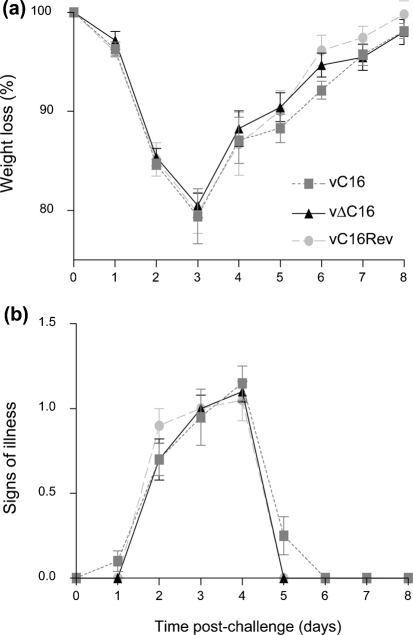
vΔC16 protects against lethal OPV challenge. Mice (*n*=5) were vaccinated subcutaneously with the indicated virus and challenged i.n. 28 days later with VACV WR (Methods). Weight change (a) and signs of illness (b) were monitored daily and data are expressed as in Fig. 5.

To characterize further the immune response to infection with vΔC16, the effectiveness of this virus as a vaccine was compared with that of the control viruses (Fig. 7). Mice were vaccinated i.d. with the indicated viruses and were challenged with VACV WR 28 days later. The challenge dose was 500 LD_50_ for BALB/c mice of this age and hence even vaccinated mice began to lose weight rapidly. Weight loss reached its maximum at day 3 post-challenge when the average weight loss of the three immunized groups (vC16, vΔC16 and vC16Rev) was 20–21 %. However, under the conditions tested there was no difference in the protection afforded by immunization with vΔC16 compared to the control viruses.

## DISCUSSION

A characterization of the C16 protein from VACV strain WR and the effects of this protein on virus replication, virulence and immunogenicity are reported. Bioinformatic analysis indicated that very closely related proteins (95–99 % amino acid identity) are encoded by five OPV species and that the gene is present, but disrupted, in three others. In addition, C16 is related to another family of OPV proteins exemplified by C4 from VACV WR, and a more distantly related group from other chordopoxviruses. C16 was expressed by all VACV and CPXV strains tested and was synthesized early during infection. This early expression profile is consistent with the analysis of the *C16L* promoter (data not shown) and a genome-wide transcriptome analysis that detected C16 mRNA by 1 h p.i. (Assarsson *et al.*, 2008). C16 is shuttled actively between the cytoplasm and nucleus but lacks a recognizable nuclear localization signal and nuclear export signal, suggesting it might be transported in a complex with another protein. The partial nuclear localization of C16 is interesting because it is one of only a few nuclear proteins encoded by VACV, which replicates in the cytoplasm. Another example is the E3 protein (Yuwen *et al.*, 1993) that binds dsRNA (Chang *et al.*, 1992) and contributes to virulence (Brandt & Jacobs, 2001). In contrast, a group of other VACV proteins that affect transcription of host nuclear factor kappa B (NF-*κ*B)-responsive genes function from within the cytoplasm (Bowie *et al.*, 2000; Bartlett *et al.*, 2002; Shisler & Jin, 2004; Chen *et al.*, 2006, 2008). There are also reports of endogenous nuclear proteins being recruited to the cytoplasm during VACV infection (Oh & Broyles, 2005).

The intracellular location of the C16 protein makes its postulated function as an IL-1ra-like protein that mediates extracellular blockade of signalling from the IL-1R (Kluczyk *et al.*, 2002, 2004) unlikely. C16 would only be in a position to modulate signalling from the IL-1R after release due to lysis of infected cells, and while this might happen very late during infection, such a function is unlikely to be its primary role given its early expression. However, there are also intracellular (ic) IL-1ra proteins, produced by differential splicing (Butcher *et al.*, 1994; Malyak *et al.*, 1998a, b) and whose functions remain poorly defined. These icIL-1ra isoforms were reported to downregulate the IL-1 pathway (Watson *et al.*, 1995; Arend *et al.*, 1998; Banda *et al.*, 2005). Moreover, the precursor form of IL-1*α*, pro-IL-1*α*, can localize to (Wessendorf *et al.*, 1993) and function within the nucleus (Cheng *et al.*, 2008) and icIL-1ra may regulate these functions. It is therefore more likely that C16 functions by mimicking icIL-1ra. Interestingly, the deerpox virus protein 054 shares 89 % amino acid identity with the secreted IL-1ra protein from *Bos Taurus* (Afonso *et al.*, 2005) and is substantially more likely to mimic extracellular IL-1ra activity than C16, but does not have a counterpart in VACV or other OPVs.

VACV already targets the IL-1 pathway by several mechanisms, indicating the importance of IL-1 against poxvirus infections. VACV restricts the formation of IL-1*β* in infected cells by expressing a caspase 1 inhibitor (Dobbelstein & Shenk, 1996; Kettle *et al.*, 1997) and blocks the function of IL-1*β* systemically by the expression of a soluble IL-1*β*R (Alcamí & Smith, 1992; Spriggs *et al.*, 1992; Alcamí & Smith, 1996). Furthermore, VACV expresses intracellular proteins A46 (Bowie *et al.*, 2000; Stack *et al.*, 2005), A52 (Bowie *et al.*, 2000; Harte *et al.*, 2003; Graham *et al.*, 2008), N1 (Bartlett *et al.*, 2002; DiPerna *et al.*, 2004; Cooray *et al.*, 2007; Graham *et al.*, 2008) and B14 (Chen *et al.*, 2006, 2008) that inhibit IL-1*α*- or IL-1*β*-induced signalling from the IL-1R that would otherwise activate NF-*κ*B.

A virus deletion mutant, vΔC16, formed a slightly smaller plaque when compared with wild-type and revertant controls, but replicated normally in the cell lines analysed and *in vivo* to reach equivalent titres at 2 days p.i. However, there was no defect in virus-induced actin tail formation, a pre-requisite for efficient cell-to-cell spread. Hence, the basis for this small plaque phenotype remains unexplained. One possibility, consistent with the presence of C16 in both the nucleus and cytoplasm, is that C16 might modulate intracellular signalling pathways and that, over several cycles of infection, replication, release and reinfection, this provides an advantage for the virus. Such a subtle difference might not be manifested during analysis of replication kinetics. A precedent for this was reported with the B14 protein of VACV WR. A mutant lacking B14 replicated normally in cell culture and *in vivo*, but produced a slightly small plaque in cell culture and an altered inflammatory response *in vivo* (Chen *et al.*, 2006). This was explained by the observation that B14 interacts with the I*κ*B kinase complex and inhibits NF-*κ*B activation (Chen *et al.*, 2008). Most VACV immunomodulatory proteins, like B14, are expressed early during infection and the early expression of C16 would be consistent with such a role.

The deletion of C16 from the VACV WR genome reduced virus virulence in a murine i.n. model (systemic infection) but not in an i.d. (local infection) model. This attenuation was characterized by significant reductions in virus-induced weight loss, signs of illness and virus titres. Although, vΔC16 virus titres in infected lungs were equivalent to control virus-infected lungs at 2 days p.i, at later times the virus titres were considerably reduced in vΔC16-infected tissues. This indicated that, in the absence of C16, the virus can replicate efficiently *in vivo*, but, in the absence of C16, virus clearance is accelerated.

Investigation of this attenuated phenotype by analysis of lymphoid cells within infected tissue indicated a difference in the number and properties of the infiltrating cells and the kinetics of their recruitment. At 3 days p.i., the number of infiltrating cells was greater after infection with vΔC16, and more CD4^+^ and CD8^+^ T cells were activated (CD69^+^). By day 7, the number of cells present and the proportion that were activated were equivalent for all viruses. However, by day 10 the total number of infiltrating cells and the number of CD4^+^ and CD8^+^ T cells were reduced after infection with vΔC16, consistent with more rapid resolution of the infection. The accelerated recruitment and activation of T cells early after infection fits with the diminished virus titres observed from day 4 p.i., and with the reduced weight loss and signs of illness. The recruitment of leukocytes to sites of inflammation is dependent on the expression of chemokines and cytokines. It follows that C16 is either directly or indirectly influencing the expression or function of such inflammatory mediators. The intracellular location of C16 suggests that it might mediate such an effect by modulating intracellular signalling pathways and this possibility is currently under investigation.

Despite the reduction of virulence of vΔC16 in the i.n. model and the difference in T-cell recruitment and activation, vΔC16 was as effective as wild-type and revertant controls in inducing protective immunity against subsequent virus challenge with an otherwise lethal dose of VACV strain WR. The vΔC16 phenotype (reduced virulence yet undiminished immunogenicity) make VACV strains lacking the *C16L* gene attractive vaccine candidates.

In summary, C16 is an intracellular virulence factor, which modulates the host response to infection. Investigation of its role in virus virulence should provide further insight into host–virus interactions and viral immunomodulation.

## References

[r1] Afonso, C. L., Delhon, G., Tulman, E. R., Lu, Z., Zsak, A., Becerra, V. M., Zsak, L., Kutish, G. F. & Rock, D. L. (2005). Genome of deerpox virus. J Virol 79, 966–977. 15613325 10.1128/JVI.79.2.966-977.2005 PMC538591

[r2] Alcamí, A. & Smith, G. L. (1992). A soluble receptor for interleukin-1*β* encoded by vaccinia virus: a novel mechanism of virus modulation of the host response to infection. Cell 71, 153–167. 1394428 10.1016/0092-8674(92)90274-g

[r3] Alcamí, A. & Smith, G. L. (1996). A mechanism for the inhibition of fever by a virus. Proc Natl Acad Sci U S A 93, 11029–11034. 8855303 10.1073/pnas.93.20.11029 PMC38278

[r4] Arend, W. P., Malyak, M., Guthridge, C. J. & Gabay, C. (1998). Interleukin-1 receptor antagonist: role in biology. Annu Rev Immunol 16, 27–55. 9597123 10.1146/annurev.immunol.16.1.27

[r5] Assarsson, E., Greenbaum, J. A., Sundstrom, M., Schaffer, L., Hammond, J. A., Pasquetto, V., Oseroff, C., Hendrickson, R. C., Lefkowitz, E. J. & other authors (2008). Kinetic analysis of a complete poxvirus transcriptome reveals an immediate-early class of genes. Proc Natl Acad Sci U S A 105, 2140–2145. 18245380 10.1073/pnas.0711573105 PMC2542872

[r6] Banda, N. K., Guthridge, C., Sheppard, D., Cairns, K. S., Muggli, M., Bech-Otschir, D., Dubiel, W. & Arend, W. P. (2005). Intracellular IL-1 receptor antagonist type 1 inhibits IL-1-induced cytokine production in keratinocytes through binding to the third component of the COP9 signalosome. J Immunol 174, 3608–3616. 15749898 10.4049/jimmunol.174.6.3608

[r7] Bartlett, N., Symons, J. A., Tscharke, D. C. & Smith, G. L. (2002). The vaccinia virus N1L protein is an intracellular homodimer that promotes virulence. J Gen Virol 83, 1965–1976. 12124460 10.1099/0022-1317-83-8-1965

[r8] Bartlett, N. W., Dumoutier, L., Renauld, J. C., Kotenko, S. V., McVey, C. E., Lee, H. J. & Smith, G. L. (2004). A new member of the interleukin 10-related cytokine family encoded by a poxvirus. J Gen Virol 85, 1401–1412. 15166422 10.1099/vir.0.79980-0

[r9] Bowie, A., Kiss-Toth, E., Symons, J. A., Smith, G. L., Dower, S. K. & O'Neill, L. A. (2000). A46R and A52R from vaccinia virus are antagonists of host IL-1 and toll-like receptor signaling. Proc Natl Acad Sci U S A 97, 10162–10167. 10920188 10.1073/pnas.160027697 PMC27775

[r10] Boyle, D. B. & Coupar, B. E. (1988). A dominant selectable marker for the construction of recombinant poxviruses. Gene 65, 123–128. 2840353 10.1016/0378-1119(88)90424-6

[r11] Brandt, T. A. & Jacobs, B. L. (2001). Both carboxy- and amino-terminal domains of the vaccinia virus interferon resistance gene, E3L, are required for pathogenesis in a mouse model. J Virol 75, 850–856. 11134298 10.1128/JVI.75.2.850-856.2001 PMC113981

[r12] Brown, C. K., Turner, P. C. & Moyer, R. W. (1991). Molecular characterization of the vaccinia virus hemagglutinin gene. J Virol 65, 3598–3606. 2041086 10.1128/jvi.65.7.3598-3606.1991 PMC241363

[r13] Buller, R. M., Chakrabarti, S., Moss, B. & Fredrickson, T. (1988). Cell proliferative response to vaccinia virus is mediated by VGF. Virology 164, 182–192. 3363864 10.1016/0042-6822(88)90635-6

[r14] Butcher, C., Steinkasserer, A., Tejura, S. & Lennard, A. C. (1994). Comparison of two promoters controlling expression of secreted or intracellular IL-1 receptor antagonist. J Immunol 153, 701–711. 8021506

[r15] Chang, H. W., Watson, J. C. & Jacobs, B. L. (1992). The E3L gene of vaccinia virus encodes an inhibitor of the interferon-induced, double-stranded RNA-dependent protein kinase. Proc Natl Acad Sci U S A 89, 4825–4829. 1350676 10.1073/pnas.89.11.4825 PMC49180

[r16] Chen, R. A., Jacobs, N. & Smith, G. L. (2006). Vaccinia virus strain Western Reserve protein B14 is an intracellular virulence factor. J Gen Virol 87, 1451–1458. 16690909 10.1099/vir.0.81736-0

[r17] Chen, R. A., Ryzhakov, G., Cooray, S., Randow, F. & Smith, G. L. (2008). Inhibition of I*κ*B kinase by vaccinia virus virulence factor B14. PLoS Pathog 4, e22 18266467 10.1371/journal.ppat.0040022 PMC2233672

[r18] Cheng, W., Shivshankar, P., Zhong, Y., Chen, D., Li, Z. & Zhong, G. (2008). Intracellular interleukin-1*α* mediates interleukin-8 production induced by *Chlamydia trachomatis* infection via a mechanism independent of type I interleukin-1 receptor. Infect Immun 76, 942–951. 18086816 10.1128/IAI.01313-07 PMC2258806

[r19] Clark, R. H., Kenyon, J. C., Bartlett, N. W., Tscharke, D. C. & Smith, G. L. (2006). Deletion of gene A41L enhances vaccinia virus immunogenicity and vaccine efficacy. J Gen Virol 87, 29–38. 16361415 10.1099/vir.0.81417-0

[r20] Cooray, S., Bahar, M. W., Abrescia, N. G., McVey, C. E., Bartlett, N. W., Chen, R. A., Stuart, D. I., Grimes, J. M. & Smith, G. L. (2007). Functional and structural studies of the vaccinia virus virulence factor N1 reveal a Bcl-2-like anti-apoptotic protein. J Gen Virol 88, 1656–1666. 17485524 10.1099/vir.0.82772-0 PMC2885619

[r21] Davison, A. J. & Moss, B. (1990). New vaccinia virus recombination plasmids incorporating a synthetic late promoter for high level expression of foreign proteins. Nucleic Acids Res 18, 4285–4286. 2377486 10.1093/nar/18.14.4285 PMC331224

[r22] DiPerna, G., Stack, J., Bowie, A. G., Boyd, A., Kotwal, G., Zhang, Z., Arvikar, S., Latz, E., Fitzgerald, K. A. & Marshall, W. L. (2004). Poxvirus protein N1L targets the I-*κ*B kinase complex, inhibits signaling to NF-*κ*B by the tumor necrosis factor superfamily of receptors, and inhibits NF-*κ*B and IRF3 signaling by toll-like receptors. J Biol Chem 279, 36570–36578. 15215253 10.1074/jbc.M400567200

[r23] Dobbelstein, M. & Shenk, T. (1996). Protection against apoptosis by the vaccinia virus SPI-2 (B13R) gene product. J Virol 70, 6479–6485. 8709286 10.1128/jvi.70.9.6479-6485.1996 PMC190684

[r24] Fenner, F., Anderson, D. A., Arita, I., Jezek, Z. & Ladnyi, I. D. (1988). *Smallpox and its Eradication*. Geneva: World Health Organization.

[r25] Goebel, S. J., Johnson, G. P., Perkus, M. E., Davis, S. W., Winslow, J. P. & Paoletti, E. (1990). The complete DNA sequence of vaccinia virus. Virology 179, 247–266, 517–563. 2219722 10.1016/0042-6822(90)90294-2

[r26] Graham, S. C., Bahar, M. W., Cooray, S., Chen, R. A.-J., Whalen, D. W., Abrescia, N. G. A., Alderton, D., Owens, R. J., Stuart, D. I. & other authors (2008). Vaccinia virus proteins A52 and B14 share a Bcl-2-like fold but have evolved to inhibit NF-*κ*B rather than apoptosis. PLoS Pathog 4, (8). e1000128 18704168 10.1371/journal.ppat.1000128 PMC2494871

[r27] Haga, I. R. & Bowie, A. G. (2005). Evasion of innate immunity by vaccinia virus. Parasitology 130 (*Suppl.*), S11–S25. 16281988 10.1017/S0031182005008127

[r28] Harte, M. T., Haga, I. R., Maloney, G., Gray, P., Reading, P. C., Bartlett, N. W., Smith, G. L., Bowie, A. & O'Neill, L. A. (2003). The poxvirus protein A52R targets Toll-like receptor signaling complexes to suppress host defense. J Exp Med 197, 343–351. 12566418 10.1084/jem.20021652 PMC2193841

[r29] Horton, R. M., Hunt, H. D., Ho, S. N., Pullen, J. K. & Pease, L. R. (1989). Engineering hybrid genes without the use of restriction enzymes: gene splicing by overlap extension. Gene 77, 61–68. 2744488 10.1016/0378-1119(89)90359-4

[r30] Jacobs, N., Chen, R. A., Gubser, C., Najarro, P. & Smith, G. L. (2006). Intradermal immune response after infection with vaccinia virus. J Gen Virol 87, 1157–1161. 16603516 10.1099/vir.0.81556-0

[r31] Kerr, S. M. & Smith, G. L. (1991). Vaccinia virus DNA ligase is nonessential for virus replication: recovery of plasmids from virus-infected cells. Virology 180, 625–632. 1989387 10.1016/0042-6822(91)90076-n

[r32] Kettle, S., Alcamí, A., Khanna, A., Ehret, R., Jassoy, C. & Smith, G. L. (1997). Vaccinia virus serpin B13R (SPI-2) inhibits interleukin-1*β*-converting enzyme and protects virus-infected cells from TNF- and Fas-mediated apoptosis, but does not prevent IL-1*β*-induced fever. J Gen Virol 78, 677–685. 9049422 10.1099/0022-1317-78-3-677

[r33] Kluczyk, A., Siemion, I. Z., Szewczuk, Z. & Wieczorek, Z. (2002). The immunosuppressive activity of peptide fragments of vaccinia virus C10L protein and a hypothesis on the role of this protein in the viral invasion. Peptides 23, 823–834. 12084512 10.1016/s0196-9781(02)00006-2

[r34] Kluczyk, A., Cebrat, M., Zbozien-Pacamaj, R., Lisowski, M., Stefanowicz, P., Wieczorek, Z. & Siemion, I. Z. (2004). On the peptide-antipeptide interactions in interleukin-1 receptor system. Acta Biochim Pol 51, 57–66. 15094825

[r35] Malyak, M., Guthridge, J. M., Hance, K. R., Dower, S. K., Freed, J. H. & Arend, W. P. (1998a). Characterization of a low molecular weight isoform of IL-1 receptor antagonist. J Immunol 161, 1997–2003. 9712072

[r36] Malyak, M., Smith, M. F., Jr, Abel, A. A., Hance, K. R. & Arend, W. P. (1998b). The differential production of three forms of IL-1 receptor antagonist by human neutrophils and monocytes. J Immunol 161, 2004–2010. 9712073

[r37] Ng, A., Tscharke, D. C., Reading, P. C. & Smith, G. L. (2001). The vaccinia virus A41L protein is a soluble 30 kDa glycoprotein that affects virus virulence. J Gen Virol 82, 2095–2105. 11514718 10.1099/0022-1317-82-9-2095

[r38] Niles, E. G. & Seto, J. (1988). Vaccinia virus gene D8 encodes a virion transmembrane protein. J Virol 62, 3772–3778. 3418784 10.1128/jvi.62.10.3772-3778.1988 PMC253521

[r39] Oh, J. & Broyles, S. S. (2005). Host cell nuclear proteins are recruited to cytoplasmic vaccinia virus replication complexes. J Virol 79, 12852–12860. 16188987 10.1128/JVI.79.20.12852-12860.2005 PMC1235867

[r40] Panicali, D., Davis, S. W., Weinberg, R. L. & Paoletti, E. (1983). Construction of live vaccines by using genetically engineered poxviruses: biological activity of recombinant vaccinia virus expressing influenza virus hemagglutinin. Proc Natl Acad Sci U S A 80, 5364–5368. 6310573 10.1073/pnas.80.17.5364 PMC384256

[r41] Parkinson, J. E. & Smith, G. L. (1994). Vaccinia virus gene A36R encodes a *M*_r_ 43–50 K protein on the surface of extracellular enveloped virus. Virology 204, 376–390. 8091668 10.1006/viro.1994.1542

[r42] Reading, P. C. & Smith, G. L. (2003). Vaccinia virus interleukin-18-binding protein promotes virulence by reducing gamma interferon production and natural killer and T-cell activity. J Virol 77, 9960–9968. 12941906 10.1128/JVI.77.18.9960-9968.2003 PMC224600

[r43] Seet, B. T., Johnston, J. B., Brunetti, C. R., Barrett, J. W., Everett, H., Cameron, C., Sypula, J., Nazarian, S. H., Lucas, A. & McFadden, G. (2003). Poxviruses and immune evasion. Annu Rev Immunol 21, 377–423. 12543935 10.1146/annurev.immunol.21.120601.141049

[r44] Shisler, J. L. & Jin, X. L. (2004). The vaccinia virus K1L gene product inhibits host NF-*κ*B activation by preventing I*κ*B*α* degradation. J Virol 78, 3553–3560. 15016878 10.1128/JVI.78.7.3553-3560.2004 PMC371086

[r45] Smith, G. L., Mackett, M. & Moss, B. (1983). Infectious vaccinia virus recombinants that express hepatitis B virus surface antigen. Nature 302, 490–495. 6835382 10.1038/302490a0

[r46] Smith, V. P., Bryant, N. A. & Alcamí, A. (2000). Ectromelia, vaccinia and cowpox viruses encode secreted interleukin-18-binding proteins. J Gen Virol 81, 1223–1230. 10769064 10.1099/0022-1317-81-5-1223

[r47] Smith, G. L., Vanderplasschen, A. & Law, M. (2002). The formation and function of extracellular enveloped vaccinia virus. J Gen Virol 83, 2915–2931. 12466468 10.1099/0022-1317-83-12-2915

[r48] Smith, G. L., Murphy, B. J. & Law, M. (2003). Vaccinia virus motility. Annu Rev Microbiol 57, 323–342. 14527282 10.1146/annurev.micro.57.030502.091037

[r49] Spriggs, M. K., Hruby, D. E., Maliszewski, C. R., Pickup, D. J., Sims, J. E., Buller, R. M. & VanSlyke, J. (1992). Vaccinia and cowpox viruses encode a novel secreted interleukin-1-binding protein. Cell 71, 145–152. 1339315 10.1016/0092-8674(92)90273-f

[r50] Stack, J., Haga, I. R., Schroder, M., Bartlett, N. W., Maloney, G., Reading, P. C., Fitzgerald, K. A., Smith, G. L. & Bowie, A. G. (2005). Vaccinia virus protein A46R targets multiple Toll-like-interleukin-1 receptor adaptors and contributes to virulence. J Exp Med 201, 1007–1018. 15767367 10.1084/jem.20041442 PMC2213104

[r51] Symons, J. A., Adams, E., Tscharke, D. C., Reading, P. C., Waldmann, H. & Smith, G. L. (2002). The vaccinia virus C12L protein inhibits mouse IL-18 and promotes virus virulence in the murine intranasal model. J Gen Virol 83, 2833–2844. 12388820 10.1099/0022-1317-83-11-2833

[r52] Tscharke, D. C. & Smith, G. L. (1999). A model for vaccinia virus pathogenesis and immunity based on intradermal injection of mouse ear pinnae. J Gen Virol 80, 2751–2755. 10573171 10.1099/0022-1317-80-10-2751

[r53] Tscharke, D. C., Reading, P. C. & Smith, G. L. (2002). Dermal infection with vaccinia virus reveals roles for virus proteins not seen using other inoculation routes. J Gen Virol 83, 1977–1986. 12124461 10.1099/0022-1317-83-8-1977

[r54] Twardzik, D. R., Brown, J. P., Ranchalis, J. E., Todaro, G. J. & Moss, B. (1985). Vaccinia virus-infected cells release a novel polypeptide functionally related to transforming and epidermal growth factors. Proc Natl Acad Sci U S A 82, 5300–5304. 3875097 10.1073/pnas.82.16.5300 PMC390555

[r55] Watson, J. M., Lofquist, A. K., Rinehart, C. A., Olsen, J. C., Makarov, S. S., Kaufman, D. G. & Haskill, J. S. (1995). The intracellular IL-1 receptor antagonist alters IL-1-inducible gene expression without blocking exogenous signaling by IL-1 beta. J Immunol 155, 4467–4475. 7594609

[r56] Wessendorf, J. H., Garfinkel, S., Zhan, X., Brown, S. & Maciag, T. (1993). Identification of a nuclear localization sequence within the structure of the human interleukin-1 alpha precursor. J Biol Chem 268, 22100–22104. 8408068

[r57] Williamson, J. D., Reith, R. W., Jeffrey, L. J., Arrand, J. R. & Mackett, M. (1990). Biological characterization of recombinant vaccinia viruses in mice infected by the respiratory route. J Gen Virol 71, 2761–2767. 2254756 10.1099/0022-1317-71-11-2761

[r58] Wolff, B., Sanglier, J. J. & Wang, Y. (1997). Leptomycin B is an inhibitor of nuclear export: inhibition of nucleo-cytoplasmic translocation of the human immunodeficiency virus type 1 (HIV-1) Rev protein and Rev-dependent mRNA. Chem Biol 4, 139–147. 9190288 10.1016/s1074-5521(97)90257-x

[r59] Yuwen, H., Cox, J. H., Yewdell, J. W., Bennink, J. R. & Moss, B. (1993). Nuclear localization of a double-stranded RNA-binding protein encoded by the vaccinia virus E3L gene. Virology 195, 732–744. 8337842 10.1006/viro.1993.1424

